# The Respiratory Tract Microbiome and Human Health

**DOI:** 10.1111/1751-7915.70147

**Published:** 2025-04-28

**Authors:** Patricia Fernández de Córdoba‐Ansón, Iván Linares‐Ambohades, Fernando Baquero, Teresa M. Coque, Ana Elena Pérez‐Cobas

**Affiliations:** ^1^ Department of Microbiology Ramón y Cajal Institute for Health Research (IRYCIS), Ramón y Cajal University Hospital Madrid Spain; ^2^ CIBER in Epidemiology and Public Health (CIBERESP) Madrid Spain; ^3^ CIBER in Infectious Diseases (CIBERINFEC) Madrid Spain

**Keywords:** human health, methods in microbiome research, microbiome biotechnology, microbiome‐based therapies, respiratory tract microbiome

## Abstract

The respiratory tract microbiome (RTM) is a multi‐kingdom microbial ecosystem that inhabits various niches of the respiratory system. While previously overlooked, there is now sufficient evidence that the RTM plays a crucial role in human health related to immune system training and protection against pathogens. Accordingly, dysbiosis or disequilibrium of the RTM has been linked to several communicable and non‐communicable respiratory diseases, highlighting the need to unveil its role in health and disease. Here, we define the RTM and its place in microbiome medicine. Moreover, we outline the challenges of RTM research, emphasising the need for combining methodologies, including multi‐omics and computational tools. We also discuss the RTM's potential for diagnosing, preventing and treating respiratory diseases and developing novel microbiome‐based therapies to improve pulmonary health.

## Graphical abstract

The respiratory tract microbiome (RTM) trains the immune system and protects against infections. Changes in the RTM are linked to various respiratory diseases. High‐throughput methodologies and computational biology can improve our understanding of the RTM, paving the way for new diagnostic tools, treatments and preventive strategies to improve respiratory health.

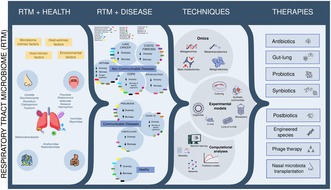

## 1 Introduction

Culture‐independent approaches and omics technologies have revolutionised microbial ecology in the past few decades, especially in investigating microbial communities associated with multicellular organisms, known as microbiomes. The microbiomes co‐evolved with their hosts driven by complex evolutionary processes and in the case of humans and their gut microbes, a parallel evolutionary history has been proposed (Groussin et al. 2020; Suzuki et al. 2022; Good and Rosenfeld 2023). In this symbiotic relationship, the gut microbiome provides several functions to human health, such as food digestion, training the immune system through metabolites production as short‐chain fatty acids (SCFAs), protecting against other microorganisms that cause disease, producing essential molecules such as vitamins B1, B2, B12, K or even more recently discovered, contributing to brain health by regulating glial functions (Wan et al. 2022; Brumfield et al. 2023; Loh et al. 2024). Similarly, other distinguishable human microbial communities are adapted to their anatomical sites, operating under different physicochemical and biological gradients, such as the oral cavity, the skin or the respiratory system (Marsland and Gollwitzer 2014).

The respiratory tract (RT) extends along all internal surfaces in touch with inhaled air, from the mouth and nostrils to the lung alveoli, comprising the upper respiratory tract (URT) that includes the oral and nasal cavities, paranasal sinuses, nasopharynx, oropharynx and supraglottic portion of the larynx and the lower respiratory tract (LRT) including the infraglottic portion of the larynx, trachea and lungs. The ear is also connected to the URT by the Eustachian tubes, so it is the case of otitis, which is mainly caused by the spread of organisms colonising the URT (Folino et al. 2020). The human respiratory tract microbiome (RTM) is a multi‐kingdom microbial ecosystem that inhabits various niches along the respiratory tract (Pérez‐Cobas, Rodríguez‐Beltrán, et al. 2023). Similarly to the co‐evolution of the gut microbiomes with their hosts, the structure of the RTM is dependent on the anatomical and functional changes occurring along the evolution of tetrapod, mammalian and human species (Pagano and Márquez 2022).

Because, for many years, it was believed that the lungs were sterile, RTM is a recent research field that has been rapidly growing. The breakthrough occurred in 2010 when the composition of the lung microbiome was described through 16S rRNA gene sequencing in patients with asthma and chronic obstructive pulmonary disease (COPD) (Hilty et al. 2010). After the lung microbiome awareness, studies based on high‐throughput sequencing technologies and culture‐independent approaches exploring the RTM association with diseases have progressively increased, principally covering cystic fibrosis (CF) (Cuthbertson et al. 2020; Cauwenberghs et al. 2024), lung cancer (Ramírez‐Labrada et al. 2020; Li, Wang, et al. 2024), pneumonia (Pettigrew et al. 2020; Pérez‐Cobas, Ginevra, et al. 2023; Drigot and Clark 2024), asthma (Barcik et al. 2020; Van Beveren et al. 2023) or COPD (Yan et al. 2022; Liang et al. 2023). Li and colleagues have recently chronologically reviewed the available literature on the RTM (Loh et al. 2024), showing an increase in the analysis of the function of the lung microbiome in host immune responses and the non‐bacterial fraction of the RT microbiome as fungi or viruses. Moreover, associated with the COVID‐19 pandemic, the lung microbiome in COVID‐19 patients also gained attention (Merenstein et al. 2022). More recently, the focus has moved to lung cancer and RTM, with growing evidence of a significant association between them (Ramírez‐Labrada et al. 2020; Tsay et al. 2021; Li, Wang, et al. 2024).

The United Nations' Sustainable Development Goals (SDGs) are focused on promoting prosperity while protecting the planet, being the SDG 3 ‘to ensure healthy lives and promote well‐being for all ages’. Several studies have shown the microbiome association with health, well‐being and disease in recent decades (Hou et al. 2022). It has also been considered ‘our last studied human organ’ (Baquero and Nombela 2012), and it is now a fundamental pillar in modern medical research and a critical element for achieving SDGs (O'Toole and Paoli 2023; Timmis et al. 2025). The microbiome has become a key component of precision medicine, which considers individual differences in molecular information (genomic, transcriptomic, proteomic and metabolomic), environment, lifestyle and other clinical data to improve medical decisions concerning treatment options (Petrosino 2018). Research towards RTM is needed to develop precision medicine strategies for respiratory diseases and incorporate the RTM into diagnostics as a point‐of‐care tool that will benefit clinics (Aogáin et al. 2022). Prior to the pandemic, COPD and lower respiratory tract infections were among the leading causes of death, exhibiting the highest age‐standardised mortality rates (GBD 2021 Causes of Death Collaborators 2024). Besides, LRT infections remained the world's most deadly communicable disease according to the WHO (The top 10 causes of death). From LRT infections, pneumonia is the leading cause of death in vulnerable populations such as children and the elderly worldwide, and it is also associated with high morbidity and short‐term and long‐term mortality in all age groups (Torres et al. 2021). Moreover, LRT infections account for a high burden of global antimicrobial resistance‐related mortality, with 1.5 million deaths estimated in 2019 attributable to antibiotic‐resistant microorganisms (Antimicrobial Resistance Collaborators 2022). The increasing antimicrobial‐resistant LRT infections are a public health threat, especially for hospitalised patients, and the role of the RTM in its dynamics remains understudied (Pérez‐Cobas et al. 2021). In this context, we present the RTM research area, a relevant, relatively new field still far from reaching the current level of knowledge of the gut, oral or skin microbiomes. We discussed the state‐of‐the‐art RTM in health and disease, the direction in which RTM research should go beyond the observational through biotechnology and the possibilities of RTM‐based therapies to fight respiratory diseases and promote health.

## 2 The Respiratory Tract Microbiome (RTM) in Health and Disease

### 2.1 Defining the Respiratory Tract Microbiome (RTM)

**FIGURE 1:**
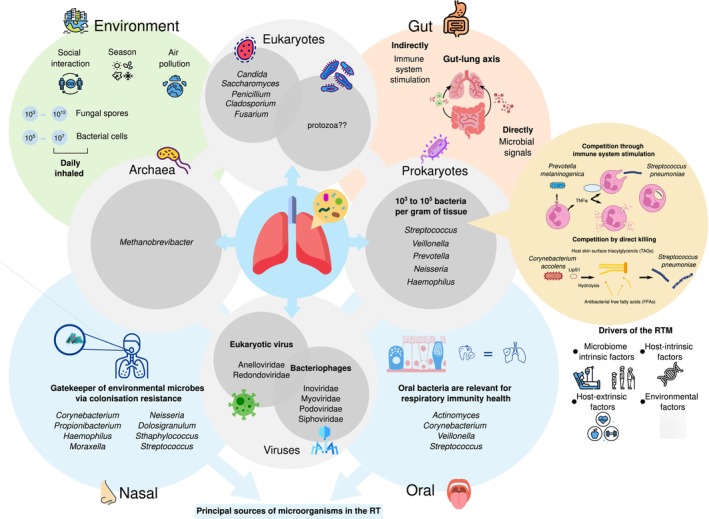
The multi‐kingdom respiratory tract microbiome (RTM) in healthy conditions. Commonly detected bacteria (and lung bacterial biomass) (Pérez‐Cobas, Rodríguez‐Beltrán, et al. 2023), fungi (Whiteside et al. 2021), eukaryotic viruses, bacteriophages (Abbas, Taylor, et al. 2019; Abbas, Young, et al. 2019; Cui et al. 2024) and archaea (Yasmine et al. 2020) are shown within grey circles around the respiratory system scheme. Protozoa (?) has been detected in the RT in individuals with respiratory conditions (Pérez‐Cobas, Ginevra, et al. 2020, 2023), while in healthy, it is unclear. The role of two beneficial bacterial species, *Prevotella melaninogenica* (competing pathogens through immune system stimulation) (Horn et al. 2022) and *Corynebacterium accolens* (competing pathogens by direct killing) for health is pictured (Bomar et al. 2016) (yellow circle). Oral and nasal microbiomes act as the primary sources of microorganisms of the RT, and they play a beneficial role in respiratory health (common bacteria of these sites are mentioned) (blue circles). The crosstalk between the gut and the RTM (gut‐lung axis) works through microbial signals between the gut microbiome and the RT or indirectly via immune system stimulation) (orange circle). As an open system, the environment also seems to impact the RTM (green). Other factors influencing RTM functioning include microbiome intrinsic factors (i.e., dysbiosis), host‐intrinsic (i.e., age, genetics) and extrinsic (i.e., medication, lifestyle), and environmental drivers (i.e., mother‐infant transmission).

The microbiomes of healthy human individuals include a spectrum of compositions and functions rather than specific profiles (Joos et al. 2025). This spectrum has been deeply analysed for the gut microbiome in different human populations (Manor et al. 2020; Sheng et al. 2024). The RT's most common taxa belong to the human‐associated phyla Firmicutes, Actinobacteria, Bacteroidetes or Proteobacteria (Figure 1). The presence of capnophilic and strict anaerobic species in the respiratory system may surprise; however, the system includes a variety of anaerobic microniches, ranging from periodontal spaces to the deep and branched epithelial crypts in the adenoids, tonsils and the pharynx (Brook 2012). However, the RTM healthy composition(s), the inter‐individual and population variability, and the stability along lifespan have yet to be uncovered. The RTM's better‐known contributions to human health are related to innate and adaptive immunity and protection against pathogen infections, two connected roles (see beneficial functions in Figure 1 and Table 1; Thibeault et al. 2021; Natalini et al. 2022; Drigot and Clark 2024; Li, Li, et al. 2024). The local impact of the RTM and its interplay with immune responses in the URT and LRT is starting to be recognised (Di Simone et al. 2023). A comprehensive review on this topic by Natalini et al. highlighted how lung microbial signatures influence the lower airway immune tone despite the constant flux of microbes acquired via microaspiration or environmental exposures (Natalini et al. 2022). Multiple investigations revealed that obligate anaerobes, mainly *Prevotella* and *Veillonella* members, are associated with improved lung infection outcomes while diminished during pneumonia (Drigot and Clark 2024). The evidence points towards subclinical lung inflammation induced by exposition to these bacteria (also present in the oral microbiome) as the protection mechanism against infections. In this regard, a mouse model showed that *Prevotella melaninogenica* protects against lung *Streptococcus pneumoniae* infection by innate immune priming, resulting in rapid pathogen clearance and improved survival (Horn et al. 2022). The recognition of *P. melaninogenica* lipoproteins by TLR2 on neutrophils triggers an intracellular signalling cascade, leading to TNFα secretion. This cytokine enhances neutrophils' pathogen‐killing capacity, improving the clearance of *S. pneumoniae* from the lungs and thus offering protective immunity. Moreover, *Corynebacterium* and *Dolosigranulum* in the nasal cavity and nasopharynx are frequently associated with reduced colonisation and infection risk of different pathogens. The protection mechanisms seem to be mediated by direct pathogen inhibition and host immune modulation (Thibeault et al. 2021; Drigot and Clark 2024). In vitro and murine model experiments revealed that *Corynebacterium accolens* inhibits *S. pneumoniae* by using the enzyme LipS1 to hydrolyse skin surface triacylglycerols in the mucosal cell membrane (Bomar et al. 2016). Afterwards, releasing antibacterial‐free fatty acids like oleic acid disrupts the *S. pneumoniae* cell membrane and prevents its growth, thereby fostering a healthier nasal microbial environment.

**TABLE 1:** Summary of the impact of microbial taxa associated with the respiratory system on health and disease, indicating effect, role, technique, model of study, and reference. Note that since this is not a comprehensive exhaustive review, only those taxa discussed in the paper are included.

Bacteria	General effect	Role or disease	Technique/Model	References
*Prevotella melaninogenica*	Beneficial	Protection against *Streptococcus pneumoniae* infection through immune system stimulation	Mouse lung co‐infection model	Horn et al. (2022)
*Corynebacterium accolens*	Beneficial	Inhibition of *S. pneumoniae* by direct killing	in vitro experiments and mouse model	Bomar et al. (2016)
*S. pneumoniae*	Harmful	Pneumonia	in vitro experiments and mouse model	Horn et al. (2022), Bomar et al. (2016)
*Prevotella oris*	Beneficial	Protection via direct microbial competition against *Moraxella catarrhalis*	in vitro culture inhibition assays	Zelasko et al. (2024)
*M. catarrhalis*	Harmful	Lower respiratory tract infection	in vitro culture inhibition assays	Zelasko et al. (2024)
*Corynebacterium propinquum*	Beneficial	Inhibition of coagulase‐negative staphylococci through siderophores	in vitro culture inhibition assays and molecular techniques	Stubbendieck et al. (2019)
Coagulase‐negative staphylococci	Harmful	Various infections	in vitro culture inhibition assays and molecular techniques	Stubbendieck et al. (2019)
*Rothia mucilaginosa*	Beneficial	Inhibition of *Pseudomonas aeruginosa* ‐induced pro‐inflammatory cytokines	in vitro 3D lung epithelial cell culture model and mouse model	Morton and Singanayagam (2022), Rigauts et al. (2022)
*P. aeruginosa* ^a^	Harmful	Infection, inflammation, tissue damage and lung disease progression in cystic fibrosis patients	in vitro 3D lung epithelial cell culture model and mouse model	Morton and Singanayagam (2022), Rigauts et al. (2022)
*Bifidobacterium bifidum*	Beneficial	Protection against influenza infection by immune system induction	Mouse model	Mahooti et al. (2019)
*Lactobacillus plantarum*	Beneficial	Protection against upper respiratory tract infections (postbiotics)	Animal models and human clinical trials	Mosca et al. (2022)
*Pediococcus acidilactici*	Beneficial	Protection against upper respiratory tract infections (postbiotics)	Animal models and human clinical trials	Mosca et al. (2022)
*Lactobacillus pentosus*	Beneficial	Protection against upper respiratory tract infections (postbiotics)	Animal models and human clinical trials	Mosca et al. (2022)
*Lactococcus lactis*	Beneficial	Improve sinus symptoms, the mucosal aspect, and promote the growth of beneficial bacteria	Human clinical trial	Endam et al. (2020)
*Lactobacillus rhamnosus*	Beneficial	As a probiotic, it kills clinic‐common pathogens, recovers respiratory microbiota, and alleviates overactive immune responses for hyperactive immunocompetent pneumonia	Mouse pneumonia model	Fu et al. (2023)
*Clostridium butyricum*	Beneficial	Reduced allergic airway inflammation and mucus secretion in allergy	Mouse model	Li, Sun, et al. (2022), Li, Zhang, et al. (2022)
*L. rhamnosus*	Beneficial	As probiotic it promotes the immune response against lung cancer development and metastases and improves the response to chemotherapy	Mouse cancer model	Le Noci et al. (2018), Le Noci et al. (2022)
*Streptococcus salivarius*	Beneficial	It is a bacteria‐producing bacteriocins with high activity against a wide range of pathogens	In vitro bacteriocin activity experiments	Hols et al. (2019)
*Bdellovibrio bacteriovorus*	Beneficial	Predation on *P. aeruginosa* , and other respiratory pathogens	In vitro predation assays	Mindt and DiGiandomenico (2022), Saralegui et al. (2022)
*Micavibrio aeruginosavorus*	Beneficial	Potent killing activity on respiratory pathogens such as *P. aeruginosa*	In vitro predation assays	Mindt and DiGiandomenico (2022)
Engineered *Mycoplasma pneumoniae*	Beneficial	High efficacy against acute *P. aeruginosa* lung infection and able to dissolve biofilms in the endotracheal tubes of patients with ventilator‐associated pneumonia	Mouse model	Mazzolini et al. (2023)
Recombinant *L. rhamnosus*	Beneficial	Boost allergen‐specific immunomodulation, prevent airway function deterioration, and promote gut microbiome equilibrium	Mouse asthma model	Spacova et al. (2020)
Engineered *B. bifidum*	Beneficial	Living nanoparticles loaded with the bacterium followed by the conjugation of an antibody efficiently target tumours, accumulate hypoxic areas and inhibit tumour growth	Mouse laryngeal cancer model	Li, Sun, et al. (2022), Li, Zhang, et al. (2022)
*Bacteriophages*	Beneficial	Improves survival and decreases bacterial load within the lungs of rats infected with methicillin‐resistant *Staphylococcus aureus*	Rat model	Prazak et al. (2019)
*S. aureus*	Harmful	Ventilator‐associated pneumonia	Mouse model	Prazak et al. (2019)
*Bacteriophages*	Beneficial	Antibiotic‐resistant *Pseudomonas* infection	Human clinical trial	Maddocks et al. (2019), Aslam et al. (2019)
*Burkholderia dolosa*	Harmful	Antibiotic‐resistant infections in lung transplant recipients	Human clinical trial	Aslam et al. (2019)

^a^ *Pseudomonas aeruginosa* is mentioned in several investigations since it is a relevant antibiotic‐resistant respiratory pathogen.

The primary sources of microorganisms in the RT are principally the oral and then the nasal microbiomes (Figure 1), and the microbes descend to the lungs through different mechanisms, such as microaspiration or dispersion (Bassis et al. 2015; Dickson et al. 2017). The microbial burden should be low in the deep lung alveoli to facilitate the lung's physiological function of exchanging oxygen and carbon dioxide. Thus, coughing, mucociliary clearance and innate and adaptive host immunity work as elimination mechanisms. The dynamic equilibrium that determines the composition of the LRTM works on the balance of immigration, elimination and the ability of the microorganisms to grow (reproduction rates) following an adapted island model proposed by Dickson et al. (2014, 2015). Subclinical aspiration of oral bacteria from the oral microbiome is relevant for respiratory immunity. Segal and collaborators defined compositional clusters (pneumotypes) by analysing the lung microbiome in healthy adults (Segal et al. 2016). Two pneumotypes were described: one enriched with oral bacteria such as *Prevotella* and *Veillonella*, higher load and inflammation markers, and another, less reactive, resembling background environmental taxa. The cellular immune responses observed in the one enriched in oral taxa suggested a role for the micro‐aspired microorganisms in regulating the basal inflammatory status. The nasal microbiome acts as a gatekeeper of environmental microbes via colonisation resistance, thus protecting and impacting the LRTM density and composition (Baker and Dickson 2024). On the other hand, the gut microbiome influences the RTM and host health through the microbial gut–lung axis, that is, the crosstalk between the gut and the RTM and/or with the immune system. Özçam and Lynch reviewed recent observations from animal and human studies that provide evidence of a bidirectional gut–lung axis, the respiratory system influencing the activity of the gut microbiome, and the fact that microbial metabolic products generated by the gut microbiome drive respiratory immunity (Özçam and Lynch 2024). One of the most outstanding examples of the gut microbiome's impact on respiratory health is the SCFAs production by gut bacteria that interact with immune cells, thus protecting against airway allergen challenge, viral influenza infection, severe lower respiratory infection or lung injury. SCFAs also shape epigenetic marks, influencing immune cell transcriptional activity and controlling airway inflammation.

On the other hand, the impact of environmental factors, such as air biotic and abiotic pollution (Wu et al. 2020; Van Kersen et al. 2022), should be determined and considered for respiratory health and disease management since the respiratory system is open and in continuous contact with the outside air. The gut microbiome is shaped by microbiome intrinsic factors (i.e., disease, age or species composition states, founder and stochastic effects), host‐intrinsic factors (those associated with anatomical differences, intestinal peristalsis, host secretions into the intestine and host genetics), host‐extrinsic factors (those associated with the lifestyle such as medication, diet and nutritional status) and environmental factors (input of regional microbial pools such as the mother‐infant transmission, household or the local environment) (Schmidt et al. 2018). Recent evidence suggests similar forces drive the RTM (Pérez‐Cobas, Rodríguez‐Beltrán, et al. 2023) (factors influencing the RTM in Figure 1).

### 2.2 The RTM and Respiratory Diseases

Research on the RTM is critical to understanding the pathogenesis and progression of communicable (or infectious) and non‐communicable respiratory diseases. In recent years, a great effort has been dedicated to understanding the association of RTM with chronic respiratory disorders, principally CF (Cuthbertson et al. 2020; Khanolkar et al. 2020; Cauwenberghs et al. 2024), asthma (Barcik et al. 2020; Cobos‐Uribe and Rebuli 2022; Van Beveren et al. 2023), COPD (Yan et al. 2022; Liang et al. 2023) or bronchiectasis (Richardson et al. 2019; Mac Aogáin et al. 2021; Mac Aogáin and Chotirmall 2022). Among the findings are, in general, altered disease‐specific microbiome composition enriched in opportunistic and/or pathogenic species, more variability and lower diversity compared to healthy individuals. However, except for CF, the most explored, more research is needed to go (beyond association) and link RTM ecological and metabolic aspects with lung function in chronic respiratory diseases (Avalos‐Fernandez et al. 2022).

In the last few years, the connection of RTM with respiratory infectious diseases, such as tuberculosis, community‐acquired and hospital‐associated pneumonia, and, more recently, COVID‐19, has been investigated, although to a lesser extent than that of chronic diseases. Even though the RTM composition in each disease is particular, general markers of RTM dysbiosis are also specific taxa enrichment as opportunistic pathogens, more composition variability, lower diversity or higher biomass compared to healthy individuals (Roquilly et al. 2019; Dickson 2021; Shah et al. 2021; Pérez‐Cobas, Ginevra, et al. 2023; Yang et al. 2024).

Respiratory infections caused by viruses are major threats to global public health. The impact of influenza A virus (FluA), influenza B virus (FluB), respiratory syncytial virus (RSV) and human rhinovirus (HRV) on the URTM was investigated in more than 300 individuals by metagenomics (Li et al. 2023). Viral type‐specific disruption was identified in the URTM, particularly in influenza infections where the composition was enriched in common bacterial respiratory pathogens (i.e., *Fusobacterium nucleatum* or *Haemophilus influenzae* ) and underrepresented in commensals such as *Streptococcus infantis* , *Streptococcus mitis* or *Corynebacterium durum*. With the COVID‐19 pandemic, considerable funding and scientific efforts have been invested in elucidating the association of respiratory, oral and gut microbiomes with this disease and its progression. Recent meta‐analyses, including several studies, found that gut and respiratory microbiomes showed composition and diversity patterns associated with COVID‐19 and disease severity, with higher significance for the gut microbiome, although the direction of causality or the mechanisms behind it are unknown (Ke et al. 2022; Merenstein et al. 2022; Reuben et al. 2023).

Regarding the role of the respiratory microbiome in infectious diseases, special attention should be paid to (i) the non‐bacterial microbial members, (ii) the identification of the resistome of the different species and the whole community and (iii) the impact of microbial migration from other microbial sources, such as the oral and gut microbiomes or the environment (Figure 1). (i) Promising studies have shown that the respiratory mycobiome is clinically relevant, being associated with disease severity, outcome and inflammatory responses in COPD (Tiew et al. 2021; de Dios Caballero et al. 2022), bronchiectasis (Mac Aogáin et al. 2018) or lung cancer (Dohlman et al. 2022). Protozoa have also been detected in the respiratory samples of patients with legionellosis, although whether they are transient or resident in the RTM remains unclear (Pérez‐Cobas, Ginevra, et al. 2020, 2023). Archaea principally from the *Methanobrevibacter* genus are part of the oral and nasal microbiomes and have also been identified in the RT, although its connection with respiratory health is not understood and needs to be further investigated (Koskinen et al. 2017; Yasmine et al. 2020; Baehren et al. 2022). The airway virome is critical for respiratory disease development and progression and virome–bacteriome profiles and specific inter‐kingdom interactions have been associated with COPD, respiratory infections and severe acute respiratory infections (Iorio et al. 2022).

(ii) Antibiotic resistance is recognised as one of the central Global Health challenges of the 21st century (Coque et al. 2023). Like the gut, the RTM can act as a source of ARGs and a site to exchange resistance‐associated elements (O'Connor and Heyderman 2023), which is impacted by the duration, spectrum, frequency of antibiotic treatments or individual's age among other variables (Pettigrew et al. 2022; Pérez‐Cobas, Rodríguez‐Beltrán, et al. 2023; Chu et al. 2024). Thus, exploring the airway resistome, including the plasmidome and other ARG‐associated elements, such as microvesicles (McInnes et al. 2020; Werner Lass et al. 2024) and bacteriophages (Leclerc et al. 2022) will shed light on ARG dynamics in the RTM and its role in antimicrobial resistance and infections caused by antibiotic‐resistant bacteria.

(iii) The impact of oral and gut microbiomes on respiratory diseases and immunity is also pivotal. This effect has been framed within landscape ecology, which examines the relationship between the spatial arrangement of organisms in different local patches and the spatial and temporal changes in local community structure (Proctor and Relman 2017). The enrichment of oral bacteria in faecal samples is a significant marker of gut bacteria depletion, and it is associated with patient outcomes consistent with gut bacteria loss (Liao et al. 2024). In the RT, low microbial diversity and dysbiosis are also associated with a higher microbial migration from the oral cavity, inflammation and decreased lung function, facilitating subsequent respiratory infections (Man et al. 2019). Increasing oral microbial diversity in idiopathic pulmonary fibrosis (IPF) predicts disease severity and death. In contrast, the oral commensal *Streptococcus mitis* is associated with the maintenance of lung function and improved survival (O'Dwyer et al. 2024). Thus, the balance between microbial immigration from the oral cavity and clearance mechanisms is key to respiratory physiology. Such a balance is variable with anatomical changes from childhood to maturity and ageing, including the key effect of emergence and loss of dentition and dental interventions.

Lipinski et al. reviewed different mechanisms by which gut microbiota impact pulmonary health via the gut‐lung axis, including alterations in the production of gut microbiota‐derived metabolites that stimulate the immune system, such as the SCFAs or L‐tyrosine, but also translocation of bacteria in acute lung injury or direct gut immune cell migration to the lungs (Lipinksi et al. 2024). A mouse‐based study described a gut‐lung axis mechanism in which intestinal antimicrobial peptide expression mediated by the gut microbiota is linked to hyperoxia‐induced lung injury and repair (Abdelgawad et al. 2023).

On the other hand, promising results suggest that the airway microbiome mediates between the environment and respiratory health, a topic that should be better investigated (Combs and Dickson 2020; Lin et al. 2023). For instance, Lin et al. showed that cigarette smoking and higher PM_2.5_ concentration were factors associated with lung function impairment via bacterial and fungal communities and exposure was associated with an enhanced inter‐kingdom interaction pattern resembling that of COPD individuals (Lin et al. 2023).

## 3 Microbial Biotechnology to Understand and Drive the RTM

### 3.1 The Molecular Biology ‘Toolbox’ to Explore the RTM

**FIGURE 2:**
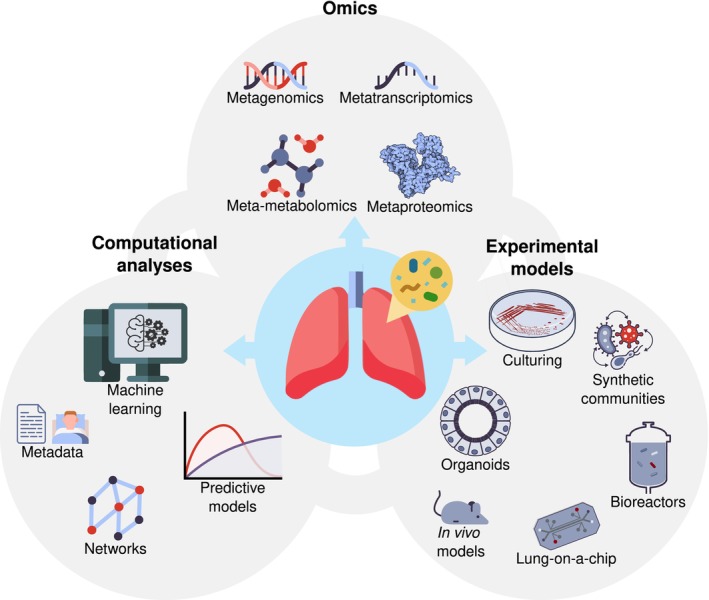
Combination of omics, computational analyses and experimental models to answer RTM‐relevant questions. Beyond marker gene sequencing, other omics (metagenomics, metatranscriptomics, metaproteomics or meta‐metabolomics) will allow in‐depth functional characterisation of the RTM. Combining derived‐omic data with comprehensive associated metadata and computational and statistical approaches, such as correlation networks or machine learning, will allow for predicting compositional clusters and microbial interaction networks. Statistically significant results, such as microbial associations, can be confirmed using in vitro and in vivo models, such as cultures, synthetic microbial communities, organoids and animals.

Culture‐independent approaches, principally marker gene sequencing and metagenomics coupled with high‐throughput sequencing technologies, have revolutionised microbial ecology. These approaches have associated human‐associated microbial communities with disease phenotypes, thus facilitating the identification of potential microbiome‐derived biomarkers, especially from the gut microbiome. Their application in the RTM area is in its infancy, and most studies are observational and based on 16S rRNA gene sequencing (Aogáin et al. 2022). The knowledge gained from more advanced research on the intestinal microbiota can serve as a basis for the steps to follow in airway microbiome investigation (Knight et al. 2018; Pérez‐Cobas and Buchrieser 2019; Pérez‐Cobas, Gomez‐Valero, et al. 2020; Walker and Hoyles 2023). Taking advantage of current technology, omic tools in an integrated multi‐omic strategy (metagenomics, metatranscriptomics, metaproteomics and meta‐metabolomics) are a fundamental first step to characterise the RTM at various complexity levels (DNA, RNA, proteins and metabolites) and its interactions (Figure 2). Thus, different omics and the multi‐omics combination have been increasingly applied in respiratory diseases such as asthma, COPD, bronchiectasis, lung fibrosis, lung transplant or lung cancer (Gao et al. 2023). From the omics, metagenomics has tremendous potential for characterising the RTM, diagnosing respiratory infections and predicting drug resistance and human host response (Charalampous et al. 2019, 2024; Diao et al. 2023). Charalampous and colleagues optimised nanopore technology for diagnosing bacterial lower respiratory infections and detecting ARGs (Charalampous et al. 2019). They validated the method with a pilot study in a real‐world critical care setting (Charalampous et al. 2024), finding a hidden infectious burden in ICU settings that was not reported by routine tests. Also, Diao et al. developed and validated a DNA‐based sequencing assay for diagnosing lower respiratory infections from bronchoalveolar lavages (Diao et al. 2023). Further translational research is crucial for promptly integrating metagenomics into clinical patient management.

Beyond a deep understanding of the composition, a thorough characterisation of the molecules, proteins and metabolites produced by RTM can give us clues about other possible local or systemic functions of the airway microbial communities. For example, Sulaiman and collaborators performed a functional characterisation of the lower airway microbiota using metagenomics, metatranscriptomics and targeted‐meta‐metabolomics (SCFAs), identifying metabolically active microorganisms that produce SCFAs which have immunomodulatory properties (Sulaiman et al. 2021). Moreover, a study that integrated sputum metagenome, metabolome, host transcriptome and proteome data shed light on critical airway microbe‐host interactions in COPD (Yan et al. 2022). Another team found that the lung microbiome can predict lung function after transplantation by analysing the bronchoalveolar microbiome, host cellular composition, metabolome and lipidome (Watzenboeck et al. 2022). Cobos‐Uribe et al. highlighted the power of multi‐omics linking the microbiome and metabolic changes (host‐microbiome metabolites exchange), significantly contributing to the pathophysiology of asthma (Cobos‐Uribe and Rebuli 2022). Integrating the microbiome omics‐derived information with the host one (DNA methylation patterns, genomics, transcriptomics, proteomics or metabolomics) will enhance our understanding of the RTM‐microbiome interplay and its physiological role.

Several techniques are available to integrate quantification in omic studies to estimate the burden of specific taxonomic groups of the RTM, such as adding spikes‐in (Pérez‐Cobas, Ginevra, et al. 2023), qPCR (Laguna et al. 2016; Pérez‐Cobas, Ginevra, et al. 2020), droplet digital PCR ddPCR (Dickson et al. 2020; Combs et al. 2021), flow cytometry or fluorescence spectroscopy (Wang et al. 2021). By combining ddPCR and 16S RNA gene sequencing, it was determined that bacterial load in the lungs and enrichment with gut‐associated bacteria predict outcomes in critically ill patients (Dickson et al. 2020). Pérez‐Cobas et al. quantified the RTM biomass in pneumonia patients through a spike‐in approach, showing that the highest bacterial and fungal loads are associated with low diversity and pathogen enrichment, suggesting high biomass as a biomarker for secondary infections (Pérez‐Cobas, Ginevra, et al. 2023). Moreover, increased lung bacterial burden (quantified by ddPCR) was predictive of chronic rejection and death in asymptomatic lung transplant recipients at 1‐year post‐transplant, suggesting that the lung microbiome could potentially be a therapeutic target for the prevention and reversal of lung transplantation chronic rejection (Combs et al. 2021).

In parallel, the development of advanced statistical and computational biology methods, including multi‐omics integration methods, has become central in microbiome omics‐based research (Deek et al. 2024). The field is moving towards artificial intelligence approaches such as machine learning or deep learning to build predictive mathematical models and determine statistical associations based on microbiome data and patients‐derived metadata (Hernández Medina et al. 2022; Figure 2). From machine learning approaches, some classical methods used for microbiome analyses are linear regression models, random forests and support vector machines, and also reduction dimension techniques are often used. It is the case of a longitudinal study that investigated the bronchoalveolar lavage fluid (BALF) microbiome and metabolome relationships between underlying lung disease and the development of chronic lung allograft dysfunction (CLAD) in post‐lung transplant individuals (Martin et al. 2024). 16S rRNA gene amplicon sequencing and untargeted LC–MS/MS metabolomics data were analysed using random forest machine learning and multivariate statistics, finding that the microbiome and metabolome after the transplant differed significantly, according to the underlying disease and that those who developed CLAD showed increased virulence metabolite production from *Pseudomonas*. Deep learning algorithms involve various artificial neural network architectures, such as fully connected neural networks or convolutional neural networks, which help deal with taxonomic abundance tables (Hernández Medina et al. 2022). Moreover, recurrent neural networks are useful to explore temporal microbiome patterns. Deep neural network models were successfully applied in a population‐based birth cohort of healthy Finnish children to investigate the relationship between the nasal microbiota and child age, considering the impact of antibiotic exposure (Raita et al. 2021). In this study, the authors illustrated the development of the nasal microbiota, highlighting shifts in specific genera during the first 2 years of life. They also found that antibiotic exposure in early infancy was associated with distinct age‐associated bacteria. Underscoring the importance of longitudinal studies, Natalini et al., through joint modelling of longitudinal and time‐to‐event data and trajectory comparisons of 16S rRNA data, investigated the association of the lung microbiome with Acute Cellular Rejection (ACR) after lung transplantation (Natalini et al. 2024). The study showed that temporal‐dependent changes in the lung microbiome are related to the development of ACR, highlighting the potential role of the microbiome in the pathogenesis or as a biomarker of this clinical complication. Other statistical techniques, such as correlation network analysis, are powerful tools for identifying putative microbial interactions and ecological patterns (Srinivasan et al. 2024). Aogáin et al. developed an approach integrating bacterial, viral and fungal data in bronchiectasis based on weighted similarity network fusion, providing a holistic view of multi‐kingdom interactions (Mac Aogáin et al. 2021). Through this multi‐omics method, the authors showed that the *Pseudomonas* interactome, rather than abundance, is associated with exacerbation risk and incorporating interaction networks improves clinical prediction models. A multi‐omic analysis, including sparse supervised canonical correlation network and random forest analyses, was used to analyse the airway microbiome, metabolome and disease outcomes in children with CF (O'Connor et al. 2022). The random forest method allowed the identification of metabolomic predictors of CF status, while the network analyses identified correlations between a traditional CF pathogen, *Staphylococcus* and nontraditional taxa in this disease, such as *Prevotella*, and a subnetwork of specific metabolomic markers. Also, in CF, through microbiome‐wide association analyses, it was shown and validated that non‐pathogenic organisms from the RT are negatively associated with lung function beyond the pathogens (Rivett et al. 2024). These studies pave the way towards novel microbiome‐based strategies targeting the interactome rather than individual pathogens in infectious diseases and other respiratory conditions. Research combining omics information with clinical data through these computational approaches allows (i) the identification of biomarkers of health and disease, (ii) the stratifying of patients based on RTM profiles and associating them to disease risk or progression, (iii) or to predict critical microbe‐microbe, host‐microbe relevant interactions and clinical outcomes.

### 3.2 Approaches to Shed Light on the RTM Ecology

Community ecology functions via a complex web of microbial connections. Different types of interactions, such as competition, predation or cooperation, have been described among RT‐related microorganisms (Pérez‐Cobas, Rodríguez‐Beltrán, et al. 2023). Identifying the key interactions in the community and understanding the molecular mechanisms that govern those interactions is the first step in learning how to manipulate or engineer them and design microbiome‐based therapies. However, in‐depth analyses of the interactions should be performed to understand disease ecology, going beyond interaction identification and microbial composition description.

Several in vitro techniques, such as co‐cultures, synthetic microbial communities or microcosms, can be applied to test putative fundamental microbial interactions in the airways (Oriano et al. 2020; Srinivasan et al. 2024) (see examples in Table 1). A shotgun metagenomic sequencing study of LRT infections showed commensal *Prevotella* spp. were depleted among early‐life infected individuals, while the pathogen *Moraxella catarrhalis* was enriched (Zelasko et al. 2024). The inhibition of *M. catarrhalis* by *Prevotella oris* was demonstrated through culture inhibition assays, suggesting a protective role via direct microbial competition. In the nasal cavity, it was also shown through co‐culture inhibition assays combined with other molecular techniques that *Corynebacterium propinquum* outcompetes coagulase‐negative staphylococci by producing siderophores that inhibit its growth. This approach revealed iron‐mediated exploitation competition as a potentially relevant protection mechanism against pathogens within the human nasal cavity (Stubbendieck et al. 2019).

Synthetic microbial consortia are promising for studying microbiome functions, metabolism, ecological dynamics, microbe–microbe and host–microbe interactions in the RT (Lemon 2020; Brüls et al. 2021; Varga et al. 2022; van Leeuwen et al. 2023; Garmendia and Cebollero‐Rivas 2024). Synthetic microbial communities of the RT could be constructed via a *bottom‐up* approach, combining well‐characterised microbial species from cultures or *top‐down* by establishing microbial communities derived from respiratory samples under controlled conditions (Garmendia and Cebollero‐Rivas 2024). Thus, microbial consortia representing the nasal microbiome represent a feasible model since few species account for 90% of diversity and many are cultivable and tractable (Lemon 2020). For example, an in vitro community based on human nasal bacteria in a tractable system led to the determination that the ecological Lotka‐Volterra model adequately illustrates the dynamics in low‐nutrient and complex (multiple interactions) (Dedrick et al. 2023).

Regarding the LRTM, a synthetic infection microbiome model of CF was successfully applied to determine the impact of antibiotics on chronic polymicrobial infections (Varga et al. 2022). The experimental model could partially recapitulate the lung microbiome observed in CF patients where oral microbes dominated the composition. The communities under clinically relevant antibiotic exposures change towards pathogen‐dominant states and enrichment of drug‐resistant species. Dental plaque and saliva microcosms have been created to research the oral microbiome ecology and its role in different diseases (Li et al. 2021; Zhou et al. 2022; Campbell et al. 2024). Microcosms of mucus were developed to investigate lung infections in CF based on artificial sputum medium and capillary tubes providing similar levels of oxygen gradient to the mucus‐plugged bronchioles one (Comstock et al. 2017). In this connection, bioreactors to study gut microbial communities have been successfully exploited to understand fermentation processes, biofilm formation, treatment testing or microbial‐community responses to antibiotic exposure (Committee on Advancing Understanding of the Implications of Environmental‐Chemical Interactions with the Human Microbiome et al. 2018; Guzman‐Rodriguez et al. 2018; Agrinier et al. 2024). Although we did not find any study indicating that it has been set up to establish a model of respiratory microbial communities, it could be a valuable tool for studying airway microbial interactions.

Computational models improve the building of synthetic microbial communities since they can predict metabolic cross‐feeding networks and the dynamics of microbial populations (McCarty and Ledesma‐Amaro 2019). A systematic computational approach, which included a lung genomic database compilation and the identification and classification of biosynthetic gene clusters, was used to describe the metabolic potential of the human lung microbiome (Semmler et al. 2024). Through a similarity network of the biosynthetic gene clusters, the authors could assign them to putative lung‐specific functions, suggesting roles in environmental adaptation, microbial competition, nutrient acquisition and communication. In CF, metabolic modelling provided new insights into the metabolic determinants of pathogen dominance observed in this disease across a range of lung nutrient conditions (Henson et al. 2019).

Other in vitro methods involving host‐derived components, such as co‐culturing with respiratory cell line cultures, organoids or lung‐on‐a‐chip microsystems, are available for testing host‐RTM hypotheses (Carney et al. 2020; Barron et al. 2021; Mahieu et al. 2024) (Figure 2, Table 1). For example, a three‐dimensional (3D) lung epithelial cell culture model helped to determine the inhibitory effect of the commensal *Rothia mucilaginosa* on the production of *P. aeruginosa* ‐induced pro‐inflammatory cytokines, supporting the role of the RTM in immune regulation (Morton and Singanayagam 2022; Rigauts et al. 2022). Wu et al. developed a lung epithelial infection model combining living material based on the co‐assembly of artificial sputum medium from CF patients able to grow 3D polymicrobial biofilms, which was used to study the impact of the antibiotic ciprofloxacin (Wu et al. 2024). The functional 3D biofilms simulate a natural biofilm's nutritional and mechanical properties and could help test complex interkingdom infection processes related to the RTM.

Airway organoids have allowed the exploration of infection capacities and cell‐derived responses to bacterial and viral pathogens such as *Mycobacterium* (Leon‐Icaza et al. 2023), respiratory syncytial virus, influenza A or COVID‐19 (Kim et al. 2022). Ex vivo lung perfusion (EVLP) is a process that enables the lung to function outside the human body. EVLP in humans and animals is a well‐suited preclinical model for translational research on chronic lung and infectious diseases (Dumigan et al. 2019; Cárdenes et al. 2021) that could be utilised to test RTM hypotheses. Recently, a protocol to understand the impact of EVLP on the lung microbiome and local inflammatory response was designed (Grando et al. 2024). An interesting work produced three‐dimensional (3D) microbiome and metabolome maps of explanted lungs from CF patients to visualise the distribution of microbes, metabolites, chemical environments and pharmaceuticals (Melnik et al. 2019). Thus, three‐dimensional organ mapping methods could be the basis for designing mechanistic studies to understand microbial interactions, RTM‐host interplay, host responses or drug metabolism.

Despite its limitations, such as significant differences in the lung's biological and anatomical structure, the mouse is the most used in vivo model to investigate the RTM (Carney et al. 2020). Diverse studies in mice have shed light on the role of RTM in innate and adaptive immune responses and their implications for respiratory health (Di Simone et al. 2023). The murine model has helped determine that the baseline lung immune tone reflects more the lung microbiome‐derived impact than the gut one (through the gut‐lung axis), a significant insight into the local RTM role for immunity (Dickson et al. 2018). However, other models have been used. For example, a rhesus macaque model showed age‐associated functional, microbial and immunological changes in the lung that partially explained the higher prevalence and severity of respiratory diseases with ageing (Rhoades et al. 2022).

## 4 Microbiome‐Based Therapies to Target Respiratory Diseases

Microbiome‐based medicine includes two critical pillars: biomarker prediction (for diagnosis, patient's stratification prior to treatment, monitoring for response to treatment) and therapeutics (prebiotics, probiotics, synbiotics, postbiotics, antibiotics, faecal microbiota transplantation or phage therapy, among others) (Gulliver et al. 2022). Several studies focus on identifying RTM features, principally diversity, composition, biomass, microbe–microbe or host–microbe interactions with the predictive power of clinical outcomes and diagnosis and prognosis biomarkers determination (some examples were developed in the previous section).

Ecology is essential to understanding the basis of disease in the microbiome era (Gilbert and Lynch 2019; Coque et al. 2023). Advancements in synthetic biology and microbiome ecology have promoted research on modulatory therapies of microbiome engineering in clinics (some examples are mentioned in Table 1). Gut microbiome‐based therapies have great potential for treating a wide range of human diseases, both intestinal and systemic; several are currently under clinical trials (Gulliver et al. 2022; Sorbara and Pamer 2022). RTM‐targeted therapies to modulate host immune lung responses or treat respiratory diseases have not yet been approved and remain in the initial research stage (Baker and Dickson 2024). Among classical approaches, antibiotics such as cotrimoxazole or erythromycin have been used to modify the RTM in IPF and non–cystic fibrosis bronchiectasis, among other diseases, with mixed results and no apparent improvements in clinical outcomes (reviewed by Chotirmall et al. (2022) Figure 3). Moreover, antibiotic usage is controversial because it has collateral effects on the microbiome, such as killing beneficial microorganisms beyond the targeted ones, reducing diversity, diminishing the colonisation resistance capacity and selecting ARGs and antibiotic‐resistant bacteria. As for the gut microbiome, alternatives to antibiotics to manipulate the RTM for therapeutics are urgently needed.

**FIGURE 3:**
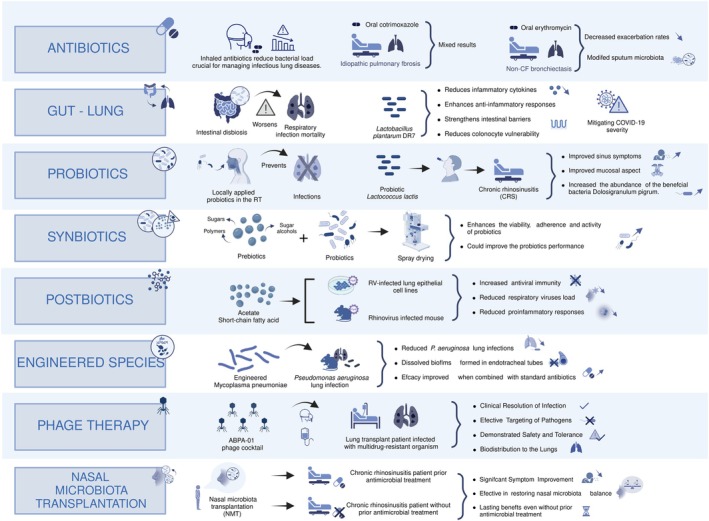
Potential microbiome‐based therapies for respiratory diseases. Diverse approaches are being tested to manipulate the RTM, targeting specific populations or eliminating RT‐associated pathogens (a few examples are shown). Some approaches can modify the RTM through antibiotics (Chotirmall et al. 2022), probiotics (Antunes et al. 2022, 2023), synbiotics (prebiotics + probiotics) (Jokicevic et al. 2021; Yuksel et al. 2023) or postbiotics (Jokicevic et al. 2021; Yuksel et al. 2023). Engineered microorganisms (Lluch‐Senar 2023) or phage therapy (Aslam 2023) can target specific microbial populations such as pathogens and/or antibiotic‐resistant species. Respiratory diseases can also be approached by stimulating the gut microbiome, which impacts the immune system (gut‐lung axis) (De Oliveira et al. 2021) or through novel approaches such as nasal microbiota transplantation (Shekhar et al. 2025).

Some gut microbiome‐based therapies, such as prebiotics, probiotics and synbiotics, have been tested to target different respiratory conditions, principally those associated with respiratory infections (Chan et al. 2020; Chotirmall et al. 2022; Debnath et al. 2022; Mindt and DiGiandomenico 2022; Mosca et al. 2022). Most of these treatments function via the gut–lung axis by administering intestinal probiotics (Chotirmall et al. 2022; Mindt and DiGiandomenico 2022; Figure 3). For example, in a mice model, the administration of the probiotic *Bifidobacterium bifidum* induced the innate and adaptive immune systems against influenza infection and treated animals showed a higher survival rate than controls (Mahooti et al. 2019). COVID‐19 disease is a good target for probiotics since the SARS‐CoV‐2 virus infects preferentially cells from the respiratory system but also affects the gastrointestinal tract. Modulating the gut microbiota through probiotics could be an important therapeutic approach with evidence of reducing inflammatory cytokines and inflammation at the systemic level, strengthening intestinal barriers and improving respiratory functioning (De Oliveira et al. 2021).

Faecal microbiota transplantation (FMT) from healthy donors has been successfully applied to patients with recurrent *Clostridioides difficile* infections (Sorbara and Pamer 2022). Despite FMT's potential for preventing or treating respiratory diseases, especially those associated with inflammation and infection, such as COPD, pulmonary emphysema, asthma, CF, bronchitis or lung cancer, it is still in the initial stages of research (Almeida et al. 2022). A mice emphysema model demonstrated that FMT and a high‐fibre diet modulating the gut microbiome attenuated the emphysema development, decreased local and systemic inflammation and protected against alveolar destruction and cellular apoptosis (Jang et al. 2020). Another study reported the case of two patients with amyotrophic lateral sclerosis (a progressive neurodegenerative disease that leads to respiratory failure) who received FMT (Yan et al. 2024). Both patients showed significant improvements in respiratory function and muscle strength, as well as an increase in beneficial microorganisms from *Bacteroides* and *Faecalibacterium* genera. Another report illustrated the case of a 95‐year‐old female patient with severe pneumonia, who post‐FMT exhibited clinical improvement, reduced sputum production and decreased diarrhoea (Zhuang et al. 2024). Clinical trials are also ongoing regarding using FMT as an adjuvant for immunomodulation to reduce COVID‐19 disease progression and to increase immunotherapy's effectiveness and lower toxicity in lung cancer patients (Almeida et al. 2022).

Another possibility is manipulating the RTM using postbiotics (inactivated microbial cells or bacterial‐derived components that confer a health benefit). Evidence in animals and patients shows the efficacy of postbiotics *Lactobacillus plantarum, Pediococcus acidilactici* and *Lactobacillus pentosus* in protecting against URT infections (Mosca et al. 2022). SCFAs are some of the most studied molecules for direct application because they directly act on microorganisms and stimulate the host immune system. For example, in a mouse model of secondary bacterial infection, acetate treatment enhanced the ability of macrophages to kill *Streptococcus pneumoniae* (Sencio et al. 2020). Although there is no consensus on the effect of SCFAs on respiratory bacterial tract infections, evidence suggests a role for specific SCFAs in stimulating host immune responses against these infections (Machado et al. 2021). More research is needed in animal models and humans through the gut and via direct application to the RT.

However, specific and locally administered probiotics in the RT might be a more targeted approach to prevent infections, as suggested for CF patients characterised by pathogen overgrowth and pulmonary exacerbations (Cauwenberghs et al. 2024). There have been some trials in humans, such as the irrigation to the nasal and sinus passages of the probiotic bacteria *Lactococcus lactis* in patients with refractory chronic rhinosinusitis (Endam et al. 2020). The probiotic improved sinus symptoms and the mucosal aspect, as well as increased the abundance of the beneficial bacteria *Dolosigranulum pigrum* . Moreover, *Lactobacillaceae* beneficial strains were formulated in a throat spray, and temporary colonisation of the throat in a metabolically active form was demonstrated, suggesting a potential for a probiotic‐spray combination in fighting viral infections (Spacova et al. 2022). Similarly, an innovative topical throat spray with beneficial lactobacilli was successfully implemented to reduce nasopharyngeal viral loads and acute symptoms in COVID‐19 patients (De Boeck et al. 2022). The optimisation of probiotics delivery and viability to the lungs to treat conditions such as CF, bronchiectasis or RT infections is being investigated, principally based on dry powder inhalations (Glieca et al. 2024; Tran et al. 2024) and nano‐particles (Fu et al. 2023). The addition of components by spray drying that can act as prebiotics (i.e., specific sugars, sugar alcohols and polymers) seems to enhance the viability, adherence and activity of RT probiotics and could improve the probiotics performance (Jokicevic et al. 2021; Yuksel et al. 2023). Probiotic‐based nanoparticles on living *Lactobacillus rhamnosus* could effectively kill various clinic‐common pathogens, increasing the overall richness and diversity of the respiratory microbiota and alleviating overactive immune responses for hyperactive immunocompetent pneumonia (Fu et al. 2023).

In allergy, the application in mice via oral and aerosol of the probiotic *Clostridium butyricum* reduced allergic airway inflammation and mucus secretion (Li, Sun, et al. 2022). Aerosol inhalation was the most effective method of probiotic administration since it restored the Th1/Th2 balance, ameliorated autophagy and inhibited the NF‐*κ*B/NLRP3 inflammasome signalling pathway. Moreover, a mice study of melanoma cancer shows that targeting the lung microbiome via aerosolisation with probiotic ( *L. rhamnosus* ) and antibiotics favours immune response against lung metastases and enhances responses to chemotherapy (Le Noci et al. 2018). Also, Noci et al. found that aerosolisation with the probiotic *L. rhamnosus* decreased the development of lung cancer in a mouse carcinogen‐induced tumour model (Le Noci et al. 2022).

Analogous to FMT, the nasal microbiome transplantation has the potential to manage and treat chronic rhinosinusitis, being successful in two studies with promising results (Mårtensson et al. 2023; Shekhar et al. 2025). Similarly, through oral microbiome transplantation, other respiratory diseases could be managed when more related to oral health in the future. Regarding postbiotics directly administered to the RT, Antunes et al. showed through in vitro experiments, mice and ex vivo treatment of patients' respiratory cells that acetate increased antiviral immunity and reduced respiratory viruses load and proinflammatory responses (Antunes et al. 2022, 2023). An interesting approach related to molecules' direct application discussed by Gosens et al. is the application of outer membrane vesicles (OMVs) containing specific beneficial products (instead of live bacteria), which could be applied to the RT or the gut (Gosens et al. 2020).

Bacteria‐producing bacteriocins (peptides of prokaryotic origin with antimicrobial properties) are another potential modulator candidate for targeting specific microorganisms (pathogens or antibiotic‐resistant species) or reshaping the RTM (Hols et al. 2019). For example, the oral bacteria *Streptococcus salivarius* appears promising to target groups of microorganisms since its bacteriocins have high activity against a wide range of pathogens (Hols et al. 2019). Predatory microorganisms that attack respiratory pathogens or modulate the RTM by eliminating specific populations are potential approaches that need deeper investigation. For example, species from *Bdellovibrio* (i.e., *Bdellovibrio bacteriovorus* ) and *Micavibrio* (i.e., *Micavibrio aeruginosavorus*) could be used as weapons since they specifically target some known pathogenic bacteria with no adverse physiological effect that has been reported upon administration in animal models so far (Mindt and DiGiandomenico 2022; Saralegui et al. 2022). Thus, further research should focus on developing therapeutics targeting the RTM (local effect) to find probiotic candidates and explore other routes, such as probiotics targeting the oral microbiome (primary source) to cure respiratory diseases (oral‐lung axis).

Engineered species can be designed to target specific lung diseases and microbiota members could produce health benefits to local or further areas through gut‐body axes. Frutos‐Grilo discussed examples of engineered species that target pathogenic bacteria, the microbiota or the immune system to treat respiratory conditions, including RT infections, lung cancer and metastasis and allergy (Frutos‐Grilo et al. 2024). In a mouse model, an engineered *Mycoplasma pneumoniae* demonstrated high efficacy against acute *P. aeruginosa* lung infections and could dissolve biofilms in the endotracheal tubes of patients with ventilator‐associated pneumonia (Mazzolini et al. 2023). Highlighting the RTM targeting, a recombinant *L. rhamnosus* probiotic designed to promote allergen‐specific immunomodulation prevented airway function deterioration and enhanced gut microbiome equilibrium in a murine asthma model (Spacova et al. 2020). Besides, in a mouse laryngeal cancer model, a delivery system using living nanoparticles loaded with *Bifidobacterium bifidum* followed by the conjugation of an antibody efficiently targeted tumours, accumulated hypoxic areas and inhibited tumour growth when used with a photodynamic and sonodynamic synergistic therapy (Li, Zhang, et al. 2022).

Clustered regularly interspaced short palindromic repeats (CRISPR)‐Cas systems can modify the genome of microorganisms. They can be programmed to eliminate specific members of microbiomes or control gene expression for metabolites and protein production (Ramachandran and Bikard 2019). Münch et al. performed a comprehensive taxonomic and functional characterisation of natural CRISPR‐Cas systems in the human microbiome and their putative targets, profiling 2.9 million CRISPR spacers (Münch et al. 2021). Interestingly, the highest CRISPR load was found in the oral habitat, which is the primary source of microbes to the RT, representing a potential target for microbiome modulation to treat respiratory diseases through the current CRISPR‐Cas applications.

Other therapeutic investigations focused on phage therapy, which has been effective in fighting RT infections in preclinical and clinical studies, especially relevant to targeting pathogenic and/or antibiotic‐resistant bacteria or destroying biofilms (Federici et al. 2021; Khosravi et al. 2024; Sithu Shein et al. 2024). Several clinical trials focus on phage therapy in adult patients with cystic fibrosis to treat *P. aeruginosa* infections (Sithu Shein et al. 2024). Other studies showed how phage therapy has been successfully applied to treat a patient with a multilobe cavitary‐resistant *Pseudomonas* infection (Maddocks et al. 2019) and in a mice model of *Staphylococcus aureus* ventilator‐associated pneumonia (Prazak et al. 2019). Moreover, phage therapy has been used in lung transplant candidates and recipients to treat antibiotic‐resistant gram‐negative infections and atypical mycobacterial infections, and it has shown a good rate of successful clinical outcomes (Aslam 2023). For example, phage therapy was used in lung transplant recipients with life‐threatening infections caused by antibiotic‐resistant *Pseudomonas aeruginosa* and *Burkholderia dolosa* (Aslam et al. 2019). When used as an adjunct to antibiotics, this treatment was well tolerated and associated with clinical improvement. Therefore, despite challenges such as safety, phage resistance or specificity, this therapy is a valuable alternative or complementary to antibiotics for treating RT infections.

## 5 Conclusions

In conclusion, although the RTM has been neglected for a long time, its role in colonisation resistance and immune system stimulation is undeniable. Recent studies have identified associations between RTM dysbiosis (altered composition, reduced diversity, pathogenic species enrichment) and various respiratory conditions. However, most aspects of the human RTM ecology must be uncovered to understand its role in human physiology and its contribution to disease. Further investigation is needed to fully understand the underlying mechanisms and translate these findings into effective therapies. Some field challenges include identifying specific targets for manipulation (i.e., specific microbial taxa, collective community features like diversity or lung bacterial burden), increasing the efficacy of respiratory microbiome‐modulating therapies, and improving our ability to deliver the actionable microbiome data to clinicians. Combining available experimental high‐throughput methodologies with advanced computational biology tools will shed light on the role of RTM in health and disease. The ultimate goal is to harness the therapeutic potential of the RTM and the development of novel RTM‐based strategies for the prevention, diagnosis, and treatment of respiratory diseases to improve respiratory health over life.

## Author Contributions

**Patricia Fernández de Córdoba‐Ansón:** writing – original draft, writing – review and editing. **Iván Linares‐Ambohades:** writing – original draft, writing – review and editing. **Fernando Baquero:** writing – original draft, writing – review and editing. **Teresa M. Coque:** writing – original draft, writing – review and editing. **Ana Elena Pérez‐Cobas:** writing – original draft, writing – review and editing, conceptualization, funding acquisition.

## Conflicts of Interest

The authors declare no conflicts of interest.

## Data Availability

Data sharing is not applicable to this article as no new data were created or analyzed in this study.
